# Trends in recurrence of primary spontaneous pneumothorax in young population after treatment for first episode based on a nationwide population data

**DOI:** 10.1038/s41598-023-39717-y

**Published:** 2023-08-18

**Authors:** Eunjue Yi, Jun Eun Park, Jae Ho Chung, Chi Bum Ahn, Eugene Chung, O Kyu Noh, Sungho Lee

**Affiliations:** 1 Department of Thoracic and Cardiovascular Surgery, Korea University Anam Hospital, 73, Koryeodae-ro, Seongbuk-gu, Seoul, 02841 Republic of Korea; 2 Department of Pediatrics, Korea University College of Medicine, Seoul, Republic of Korea; 3 Biomedical Engineering Research Center, Asan Medical Center, Seoul, Republic of Korea; 4 Department of Linguistics, Korea University, Seoul, Republic of Korea; 5 Department of Radiation Oncology, Ajou University School of Medicine, 164 Worldcup-ro, Yeongtong-gu, Suwon, 16499 Republic of Korea; 6 Department of Biomedical Informatics, Ajou University School of Medicine, Suwon, Republic of Korea; 7 Office of Biostatistics, Ajou Research Institute for Innovative Medicine, Suwon, Republic of Korea

**Keywords:** Diseases, Health care

## Abstract

The aim of this study is to identifying post treatment recurrence rates in pneumothorax patients under 35 and without any comorbidities according to the treatment types, gender, and age categories based on nationwide population data. Clinical information of pneumothorax patients was extracted from the Korean National Health Insurance Service (NHIS) database between January 2002 and December 2020. Enrolled patients were categorized into two groups; (1) Group I, those who underwent conservative management including pain relief, oxygen therapy, and closed thoracostomy, and (2) Group II, surgical intervention. Recurrence rates were compared according to age, gender, and type of treatment. Surgical intervention was performed in 25.6% patients as first treatment. The overall recurrence rate was 20.3%. Male patients showed a higher 5-year recurrence rate than female (20.8% vs. 10.9%, p < 0.001). Those with conservative management showed lower 5-year recurrence rates than those with surgical treatment (7.9% vs. 23.7%, p < 0.001). The 5-year recurrence rates of patients aged 14≤, and < 20 was higher than other age groups (29.2% vs. 4.5 and 11.9%, p < 0.001). Surgical intervention, male gender and aged under 20 showed association with higher recurrence rates.

## Introduction

Spontaneous pneumothorax is defined as air collection in pleural space without any traumatic forces. Bimodal curve was noted in age distributions, one was between 15 to 34, and the other was aged over 55–65^1–3^. The term ‘primary’ can be added when no underlying pulmonary pathologies are found, which is common in the first peak population with reported annual incidence of 7.4 men and 1.2 women per 100,000 individuals^4^.

Although there are well established treatment guidelines^5–8^, no consideration relating with age differences have been reflect. Current treatment process for young SP patients otherwise healthy tended to be largely relied on clinician’s customization^9,10^. Methodologies for estimating severity of pneumothorax such as calculating the amount of collected air in the thorax or the size of bullae on the pleural surface were mostly derived from imaging studies of adults^11^. Therapeutic algorithms suggest non-surgical management including observation, oxygen inhalation, needle aspiration, and drainage using a small- or large-bore catheter for first episodes^5,6,8^, however, surgical intervention would be justified considering high recurrence rates reported up to 50%^4,5^.

Because the main purposes of PSP treatment are re-expansion of the collapsed lung and recurrence prevention^8^, initial surgical intervention for correcting the pathologic status directly may be attractive. This would remove the potential risk factors of air leakage or recurrence, such as bullae or blebs, thereby significant reduction in recurrence rates and hospital stays^12^.

Nevertheless, surgical outcomes in young PSP patients seemed to be controversial. Several researches proposed that the prevalence of recurrence tends to increase in younger patients despite surgical treatment^13–15^. Age under 18 was often referred as one of risk factors for recurrence after surgery. Other studies recommended Video-assisted thoracoscopic surgery (VATS) to be first-line therapies for children^10^, occasionally confined to cases that predisposing factors exist^9^.

The ambiguity in PSP treatment could be due to the poor understanding of precise pathogenesis. The visible emphysema-like changes (ELCs), called bullae or blebs, have been considered to be causes of intrapleural air collection through a rupture site^16^; but this hypothesis has have never been proven^8^. Moreover, the ELCs are not always detectable^17^. A unique pleural fibrosis pattern with fibroblastic foci at the margin of pleura and parenchyma in young pneumothorax patients was noted^18^. There is a possibility of diffuse air passage through invisible pores lining a weak area of visceral pleura^19^.

The incomplete growth process can be one reason for the increase in recurrence rate. The rapid longitudinal growth during puberty was reported to induce inflating pressure and, therefore, to reduce compliance^20^. Delayed puberty was frequently observed in young PSP patients to which the risk of recurrence after the surgical intervention can be attributed^21^. Because puberty is associated with early acceleration and late deceleration of growth velocity^22^, surgery can induce the vulnerable environment for recurrence by modifying the premature lung parenchyma. recent studies have suggested the possibility of changing paradigms to minimize treatment invasiveness^23,24^

Based on nationwide population data, we aimed to estimate the recurrence rate after surgery in young PSP patients according to age distribution; children, adolescence, or young adult, and gender Comparison with recurrence tendency of patients who did not undergo surgery was also performed. The aim of this study is to understand trends of recurrence pattern in young PSP patients who were in difference developing periods according to the gender and treatment types.

## Materials and methods

### Data sources

For a nationwide population-based study, we extracted data from the Korean National Health Insurance Service (NHIS) cooperation system between January 2002 and December 2020 (customized data number: NHIS-2021-1-808). The customized data of this study was extracted and provided according to the workflow of the National Health Insurance System Sharing Service (https://nhiss.nhis.or.kr/bd/ab/bdaba032eng.do). Health insurance claim data from this system is stored and managed by classifying a single claim statement into several tables such as general statement details, disease details, treatment details, and medical institution prescription details, and it includes the statement identification number information as a common variable, enabling linkage between tables. The general statement detail table includes basic information of the patient (gender, age, insurance type), disease (codes of major diagnosis/first auxiliary diagnosis codes), treatment information, and information on medical expenses.

Korean NHIS covers over 98% of domestic people for medical services. It is a single payer system, from which, medical providers can be reimbursed for their services. Patient who had been treated in any kinds of hospitals were enrolled in this system with the International Statistical Classification of Diseases and Related Health problems, 10th revision (ICD-10). The therapeutic records were detected using specific treatment relating diagnosed diseases.

### Study population and categorization

Patient registry who were given ICD-10 codes relating with spontaneous pneumothorax Korean NHIS between January 2002 and December 2020. Associating ICD codes were J93 (pneumothorax), J93.0 (spontaneous tension pneumothorax), J93.1 (other spontaneous pneumothorax), J93.8 (other pneumothorax) and J93.9 (pneumothorax unspecified). Pneumothorax relating with trauma (S27.x) was not involved.

The incidence trends of spontaneous pneumothorax typically showed bimodal age-distribution, first peak at age between 15 to 34 and the other age beyond 60 s^1–3^. Primary spontaneous pneumothorax generally affects young healthy population, therefore we enrolled first peak of age-distribution in this study. Patients who were (1) aged ≥ 35 when pneumothorax was first diagnosed, or assigned with other codes associated with pulmonary diseases before the first diagnosis of pneumothorax or simultaneously, such as (2) with congenital pulmonary disease, (3) diagnosed as having pneumonia, (4) tuberculosis, and (5) with cancers were not included for excluding any secondary causes. Relating ICD codes (relevant diseases among A, B, C, J M, O P codes) were used (Supplementary Table S1).

Enrolled patients were categorized into two groups according to therapeutic types for their first pneumothorax. Group I patients underwent conservative management, including observation, pain relief, oxygen therapy, and closed thoracostomy. Group II patients underwent surgical intervention. The recurrence rates of the two groups according to age distributions and gender were evaluated and compared. This study was approved by the Institutional Review Board (IRB) of the Korea University Anam Hospital (IRB number: 2021AN0334) and was performed in a manner consistent with the institution’s ethical policies and standards.

### Definition of recurrence

Healthcare Insurance Claim Data contains unique identifiers, enabling patient differentiation and facilitating the creation of patient follow-up information. Recurrence of pneumothorax was defined when the diagnostic codes (J93, J93.1, J93.8 and J93.9) were assigned after the first treatment until the data extraction time (2022.05) due to: (1) readmission with pneumothorax diagnostic codes or (2) more than three outpatient visits for pneumothorax within two months in any time of follow-up periods.

### Treatment types for first pneumothorax

We identified therapeutic options, including simple observation, pain relief, oxygen inhalation, closed thoracostomy, and surgical intervention. Surgical intervention included video-assisted thoracoscopic surgery, usually wedge resection for bullae. Treatment modalities other than surgery were classified as conservative management. Since Health insurance claim data assign specific codes to each treatment action and prescription for payment, data were extracted using those. For identifying treatment, codes for oxygen therapy (C1000021), closed thoracostomy (T006405), Pleurodesis (T001080, T001081 and T006394), and surgery (T006217 and T006219). Patients who were assigned by codes for surgery were classified as Group II, and others as Group I.

### Classification according to age categories

Patients were divided into three age categories: (1) age under 14 years, (2) age ≥ 14 years and < 20 years, and (3) ≥ 20 years. Age 14 is considered to be the age of the last growth spurt in Korean children^25^, and age 20 is the border between adolescence and adulthood in the WHO classification^26^. Patient distribution according to age categories is described in Table 2.

### Statistical analysis

The cumulative incidences of recurrence were calculated using the competing risk method regarding any death event as a competing risk. Gray's test estimated the differences in cumulative incidences between groups. The comparison among groups was performed using the chi-squared test for categorical variables and the student t-test for continuous variables. All statistical analyses were performed using R 3.0.2 software (www.r-project.org).

### Ethics approval and consent to participate

This study was approved by the Institutional Review Board (IRB) of the Korea University Anam Hospital (IRB number: 2021AN0334). The necessity for acquiring written informed consent from the patients included in this study was waived because this was retrospective study and any individual information was not identifiable in the text. The study used de-identified secondary health insurance claim data collected and distributed by NHIS under the government Ministry of Health and Welfare. As the data is available upon request to NHIS (www.nhis.or.kr), the study received a waiver of Ethical approval from Yonsei University Health System, Severance Hospital, Institutional Review Board (Wavier approval number: 4–2021-1375). Moreover, the informed consent was waived as the data is provided by NHIS. The waiver was approved based as Enforcement Rules Of The Bioethics and Safety Act, Republic of Korea [Article 15 (Deliberation on Human Subjects Research Projects) (1) A person who intends to conduct a human subjects research project shall prepare a research plan and submit it for deliberation by the competent IRB before commencing such human subjects research project. (2) Notwithstanding paragraph (1), a research project may be exempted from deliberation by the competent IRB, if a risk to human subjects of research and the general public is insignificant and the research project meets the standards prescribed by Ordinance of the Ministry of Health and Welfare after deliberation by the National Committee].

## Results

### Patient demographics

**Figure 1:**
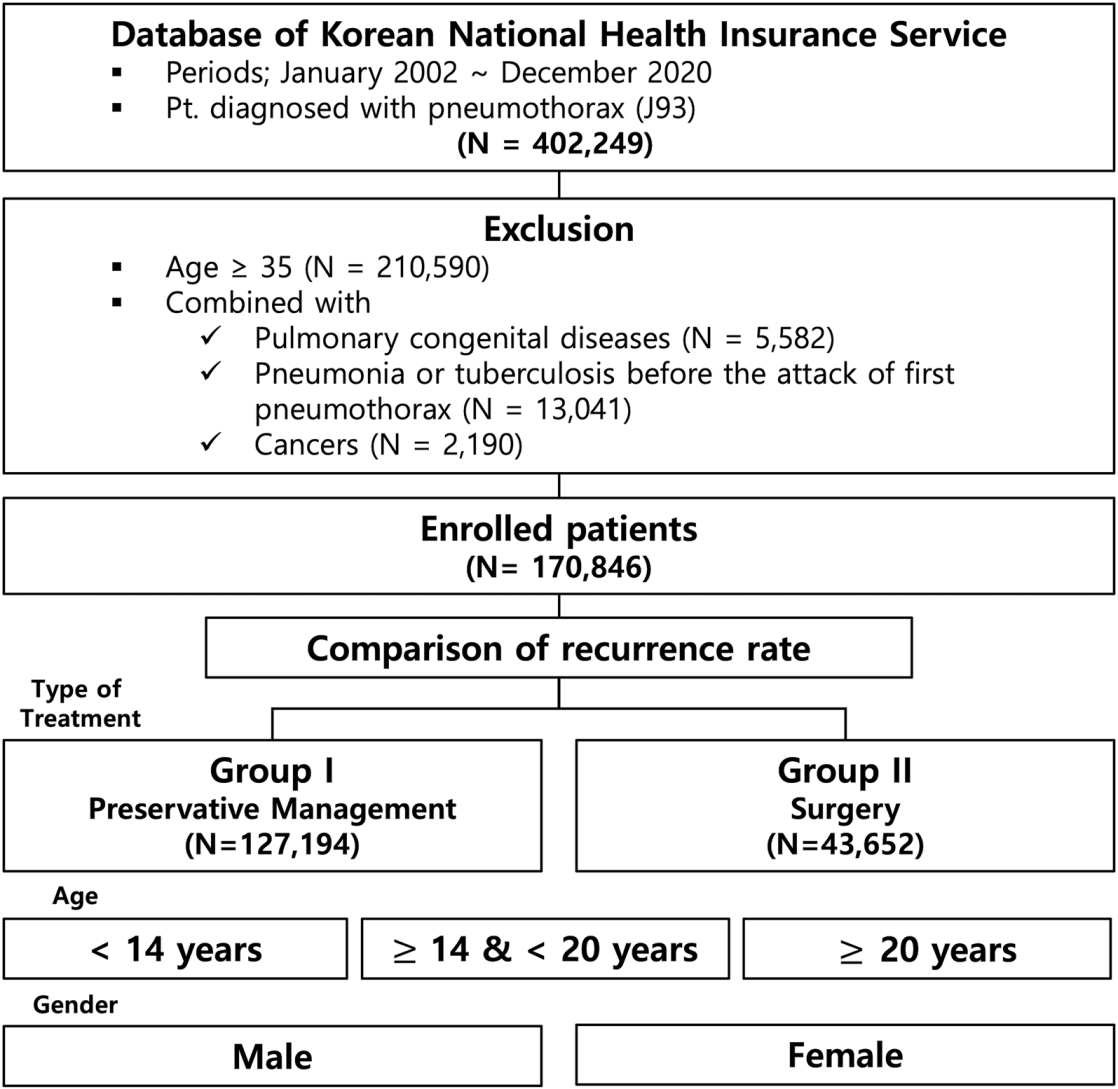
Flowchart for patient enrollment; patient data were derived from Korean NHIS.

Among a total of 402,249 cases of spontaneous pneumothorax, 191,659 patients under 35 years of age were identified. Patients with congenital pulmonary disease (N = 5582), diagnosed as having pneumonia or tuberculosis before the attack of pneumothorax (N = 13,041), and with cancers (N = 2190) were excluded (Fig. 1). Patient demographics were described in Table 1.

**Table 1:** Patient characteristics. Patients were distributed according to the year of diagnosis, gender and age groups. Population with conservative management were significantly higher than those of surgery (p < 0.001). Male.

Variables	Group I (conservative)	Group II (surgery)	Total	*p*-value
	(N = 127,194)	(N = 43,652)	(N = 170,846)	
	N (%)	N (%)	N (%)	
Year of diagnosis				< 0.001
2002–2005	32,939 (26.0)	8,049 (18.4)	40,988 (24.0)	
2006–2010	38,209 (30.0)	13,469 (30.9)	51,678 (30.2)	
2011–2015	33,357 (26.2)	12,997 (29.8)	46,354 (27.1)	
2016–2020	22,689 (17.8)	9,137 (20.9)	31,826 (18.6)	
Gender				< 0.001
Men	106,771 (83.9)	39,709 (91.0)	146,480 (85.7)	
Women	20,423 (16.1)	3,943 (9.0)	24,366 (14.3)	
Age				< 0.001
	21.3 ± 6.8	21.0 ± 5.0	21.2 ± 6.4	
Group				< 0.001
< 14	8,893 (4.6)	638 (1.5)	9,531 (3.8)	
≤ 14, < 20	57,386 (46.3)	25,753 (59.0)	83,139 (49.6)	
≤ 20	60,915 (49.1)	17,261 (39.5)	78,176 (46.6)	
Recurrence				< 0.001
	23,891 (18.8)	10,852 (24.9)	34,743 (20.3)	

**Figure 2:**
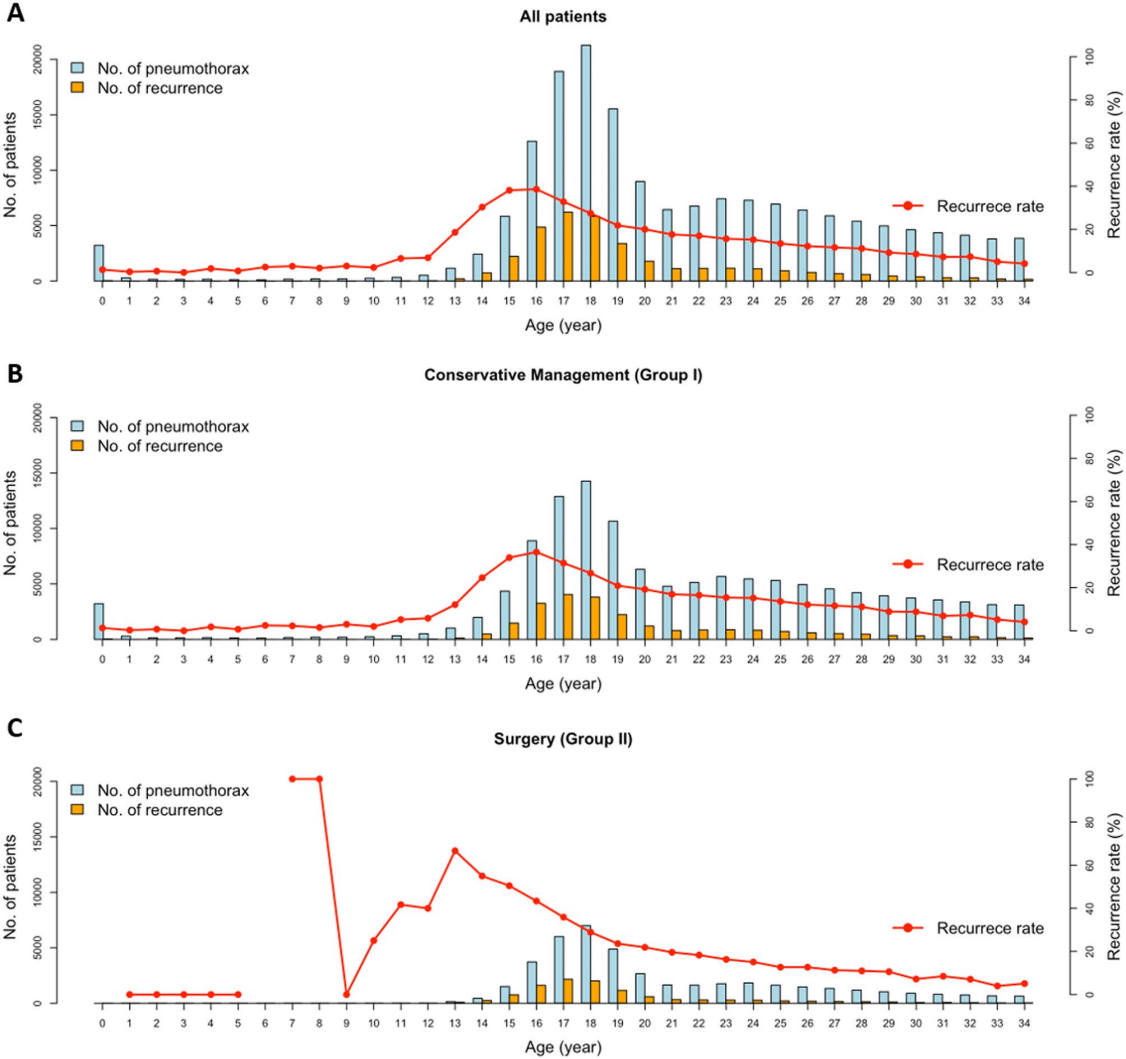
Distribution of enrolled PSP patients with proportion of recurrence according to ages (celeste bar indicates number of patients and yellow means that of recurred ones at specific ages). Traces of recurrence rates are marked with red dots and lines. (**A**) Frequencies of all patients. (**B**) Those of Group I and Group II.

**Figure 3:**
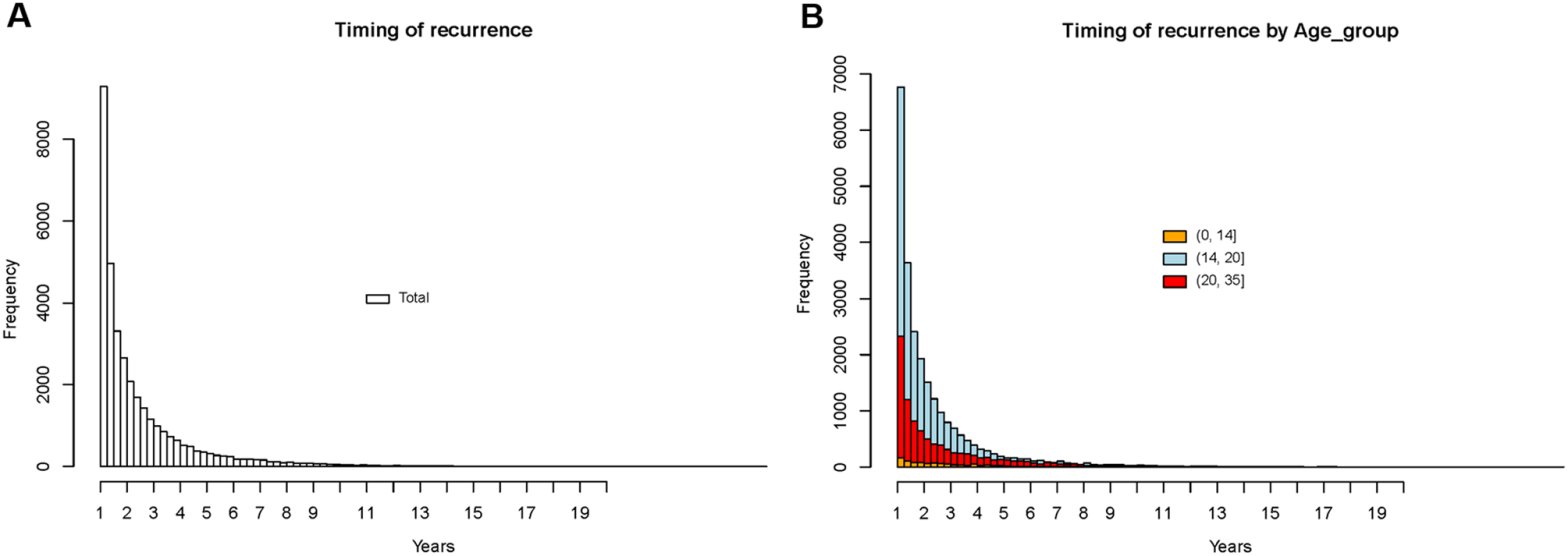
(**A**) Timing or recurrence as the year after 1st diagnosis of pneumothorax. Most of the recurrence cases tended to occur in 4–5 years after 1st episodes. (**B**) Timing of recurrence as the year after 1st diagnosis of pneumothorax according to the age categories. Trends of recurrence seemed to be similar among age categories.

**Figure 4:**
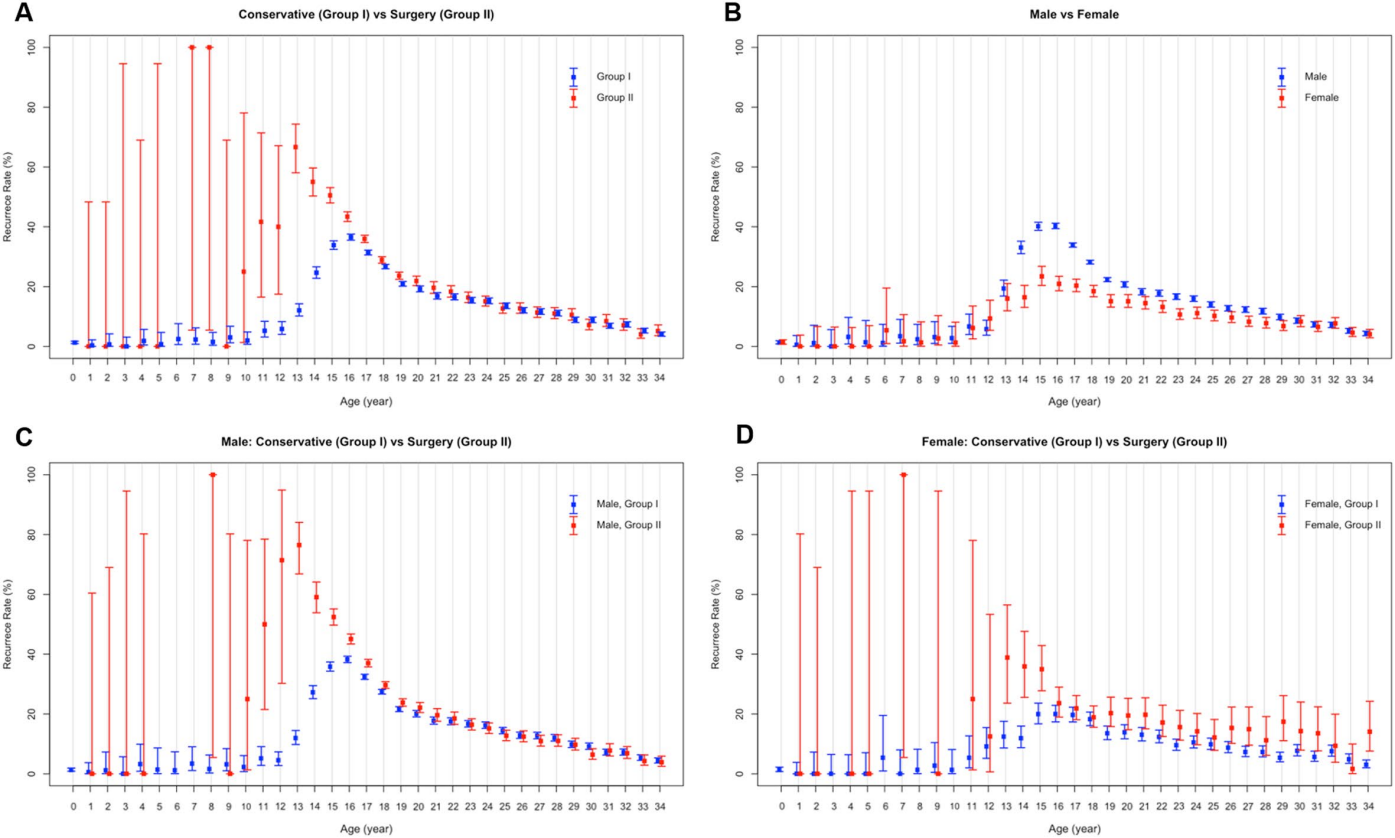
High-low-closed charts according to the treatment groups and gender. (**A**) Comparison of patient distribution and recurrences between Group I and Group II (blue, Group I, and red, Group II). Ages under 20 showed markedly larger recurrence rates. (**B**) Patient distribution by gender (blue, male and red, female). The recurrence rates of men tended to be higher than those of women. (**C**) Comparison of Group I and II in male population. Group II showed higher recurrence rates especially in those under 20. (**D**) Comparison between treatment groups in females. Group II showed higher recurrences, similar to males.

A total of 170,846 patients were enrolled in this study. Men comprised 146,480 (85.7%) and women, 24,366 (14.3%). There were significantly more men in Group II than Group I (*P* < 0.001). A flowchart for patient categorization is shown in Fig. 1. Age distribution for first pneumothorax diagnosis and its recurrence rates are illustrated in Fig. 2. Timing of recurrence after 1st episodes by year were described in Fig. 3, and high-low-closed charts according to the treatment groups and gender, in Fig. 4.

### Overall and 5-year recurrence rates

**Figure 5:**
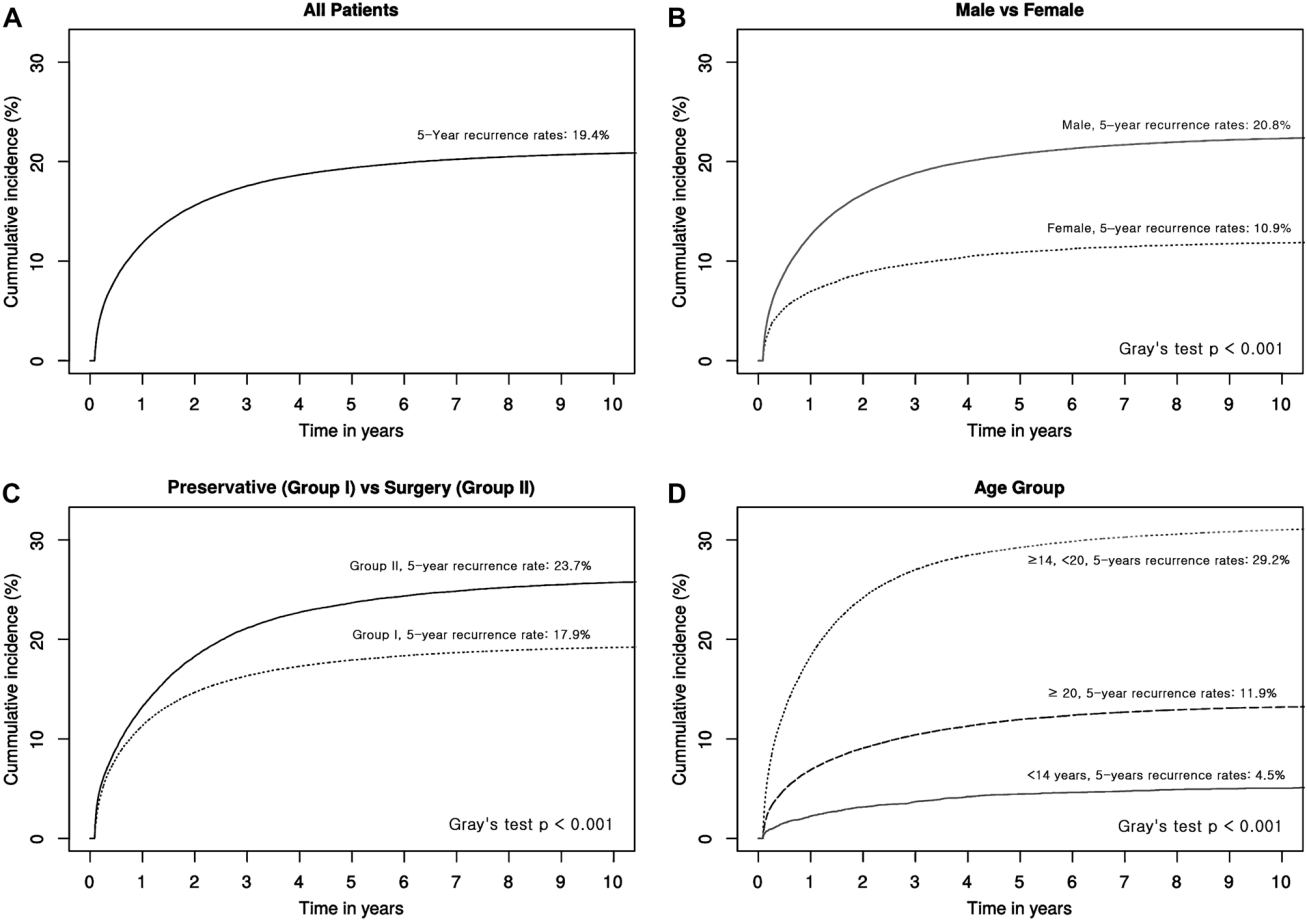
Cumulative recurrence curves. (**A**) Recurrence curves of all patients. The 5-year recurrence rates were estimated as 19.4%. (**B**) Recurrence rates by gender. The 5-year recurrence rates of male were significantly higher than those from female (*p* < 0.001). (**C**) Recurrence rates curves between Group I and II. The 5-year recurrence rates of Group II were significantly higher than those of Group I (p < 0.001). (**D**) Curves of recurrence rates according to the age categories. The 5-year recurrence rates of age ≤ 14 and < 20 were significantly higher than other ages categories (*p* < 0.001).

The overall recurrence rates were 20.3%, and the 5-year rates, 19.3%. The 5-year recurrence rates of male patients were higher than those of females (20.8% vs. 10.9%, p < 0.001), and the 5-year recurrence rates of Group I patients were significantly lower than those of Group II patients (17.9% vs. 23.7%, p < 0.001). According to the age categories, patients aged ≤ 14 years, and > 20 years showed significantly higher recurrence rates compared with the other age groups (29.2% vs. 4.5% and 11.9%, p < 0.001) (Fig. 5).

### Comparison of Group I and II

**Figure 6:**
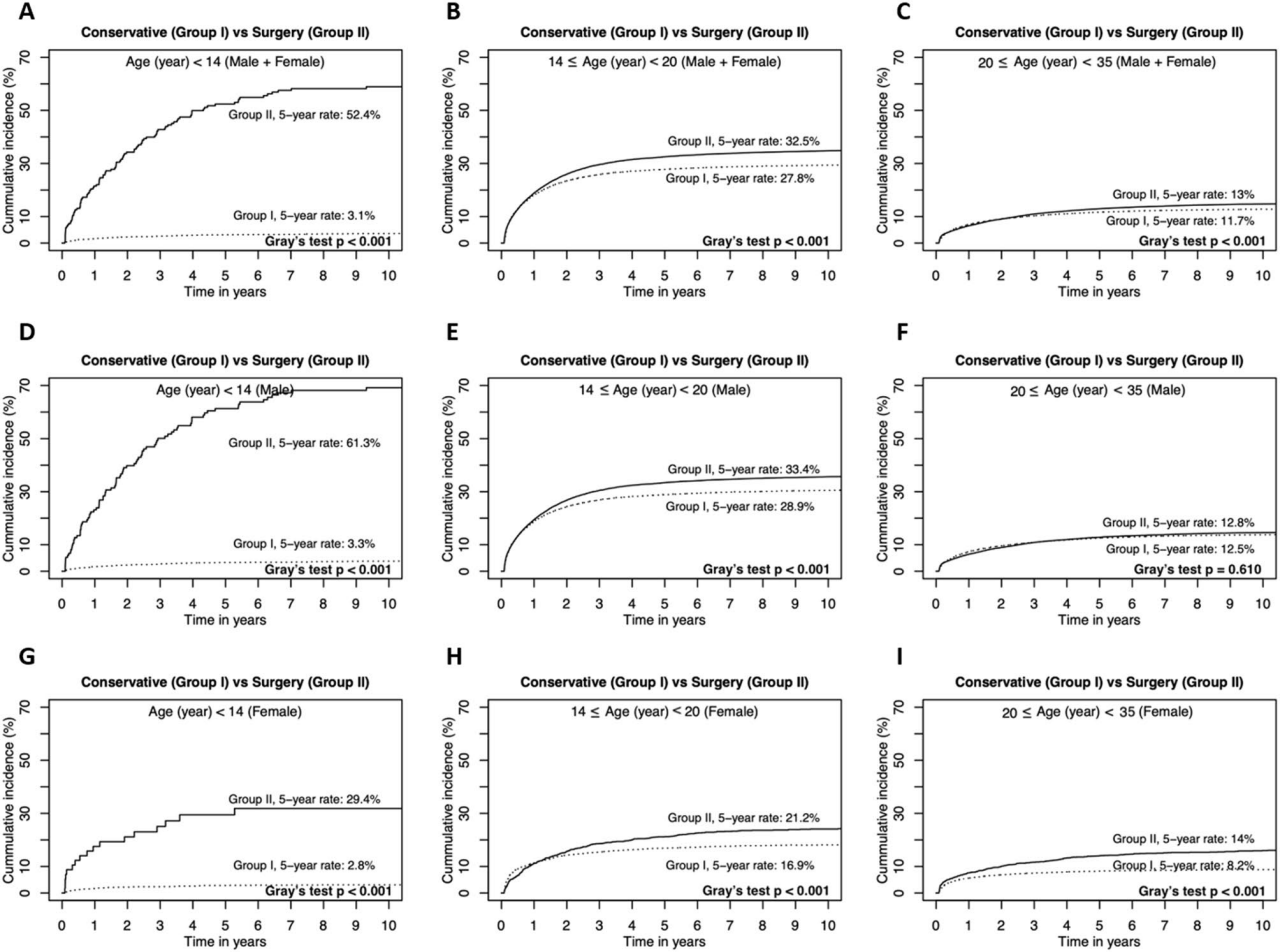
Comparison of 5-year recurrence rates between Group I and Group II according to age categories and gender. Group II showed significantly higher recurrence rates in all age and treatment groups except age categories ≥ 20 in male. (**A**) Comparison of 5-year recurrence curves between Group I and Group II. (**B**) Those in aged ≤ and > 20. (**C**) Those in aged ≥ 20. (**D**) Comparison of 5-year recurrence rates between Group I and Group II in male patients. (**E**) Those in aged ≤ and > 20. (**F**) Those in aged ≥ 20. (**G**) Comparison of 5-year recurrence rates between Group I and Group II in female patients. (**H**) Those in aged ≤ and > 20. (**I**) Those in aged ≥ 20.

The cumulative recurrence rates were compared between Group I and Group II (Fig. 6) according to the age categories and gender respectively. Group II showed higher 5-year recurrence rates in all age categories except adult male (Fig. 6F. 12.5 vs 12.8%, *P* = 0.610).

### Comparison among age categories

Table 2 shows the overall recurrence rates by age categories. The 5-year recurrence rates of ages ≥ 14 and < 20 were significantly higher than the other age categories (< 14, and ≥ 20) in all patients, regardless of gender and Group I classification (Supplementary Fig. S2A,B,D,E,G,H). In Group II, age under 14 categories showed over 50% recurrence rates, showed higher recurrence rates than other age categories and gender (Supplementary Fig. S2C,F,I, *P* < *0.001*). Adjusted hazard ratio (HR) of recurrence according to the age categories relating with gender and treatment groups were described in Table 3.

**Table 2:** Patient demographics with recurrence rates according to the age categories; ages under 14, ages ≥ 14 as well as < 20, and ≥ 20.

Variables	Group I (conservative)			Group II (surgery)			Total			*p*-value
	(N = 127,194)			(N = 43,652)			(N = 170,846)			
Age categories	< 14	≥ 14, < 20	≥ 20	< 14	≥ 14, < 20	≥ 20	< 14	≥ 14, < 20	≥ 20	
	(N = 8,893)	(N = 57,386)	(N = 60,915)	(N = 638)	(N = 25,753)	(N = 17,261)	(N = 9,531)	(N = 83,139)	(N = 78,176)	
	N (%)	N (%)	N (%)	N (%)	N (%)	N (%)	N (%)	N (%)	N (%)	
Year of diagnosis										< 0.001
2002–2005	1,744 (19.6)	11,986 (20.9)	19,209 (31.5)	90 (14.1)	3,904 (15.2)	4,055 (23.5)	1,834 (19.2)	15,890 (19.1)	23,264 (29.8)	
2006–2010	2,488 (28.0)	17,605 (30.7)	18,116 (29.7)	231 (36.2)	7,925 (30.8)	5,313 (30.8)	2,719 (28.5)	25,530 (30.7)	23,429 (30.0)	
2010–2015	2,551 (28.7)	17,199 (30.0)	13,607 (22.3)	186 (29.2)	8,332 (32.4)	4,479 (25.9)	2,737 (28.7)	25,531 (30.7)	18,086 (23.1)	
2016–2020	2,110 (23.7)	10,596 (18.5)	9,983 (16.4)	131 (20.5)	5,592 (21.7)	3,414 (19.8)	2,241 (23.5)	1,6188 (19.5)	13,397 (17.1)	
Gender										< 0.001
Male	6,241 (70.2)	52,017 (90.6)	48,513 (79.6)	503 (78.8)	23,874 (92.7)	15,332 (88.8)	6,744 (70.8)	75,891 (91.3)	63,845 (81.7)	
Female	2,652 (29.8)	5,369 (9.4)	12,402 (20.4)	135 (21.2)	1,879 (7.3)	1,929 (11.2)	2,787 (29.2)	7,248 (8.7)	14,331 (18.3)	
										< 0.001
Mean age	10.7 ± 4.1	17.6 ± 1.4	26.8 ± 3.9	13.3 ± 2.0	17.7 ± 1.4	26.2 ± 3.7	11.0 ± 4.0	17.7 ± 1.4	26.6 ± 3.9	
										< 0.001
Recurrence	728 (8.2)	16,025 (27.9)	7,138 (11.7)	352 (55.2)	8,287 (32.2)	2,213 (12.8)	1,038 (10.9)	2,4312 (29.2)	9351 (12.0)

**Table 3:** Adjusted Hazard ratio after 1st episode of pneumothorax according to age categories. Gender and treatment groups were contained in Gray’s test for calculating hazard ratio.

Variables	Age categories									Total	
	< 14			≥ 14, < 20			≥ 20				
	HR (95% CI)		p-value	HR (95% CI)		p-value	HR (95% CI)		p-value	HR (95% CI)	p-value
Age											
< 14										1.5 (1.41–1-60)	< 0.001
≥ 14, < 20										2.61(2.55–2.68)	< 0.001
≥ 20										Ref	
Gender											
Male	Ref			Ref			Ref			Ref	
Female	0.44 (0.37–0.53)		< 0.001	0.58 (0.525–0.61)		< 0.001	0.73 (0.68–0.77)		< 0.001	0.63 (0.61–0.66)	< 0.001
Treatment											
Group I (conservative)	Ref			Ref			Ref			Ref	
Group II (surgery)	6.03 (5.29–6.86)		< 0.001	1.16 (1.13–1.19)		< 0.001	1.07 (1.02–1.12)		< 0.001	1.18 (1.15–1.21)	< 0.001

*HR* Hazard ratio, *CI* confidential interval.

### Comparison between genders

Comparison of cumulative recurrence rates between male and female was performed according to the age categories and treatment groups (Supplementary Fig. S3). The recurrence rates of male were higher than those from female in all age categories and treatment groups except in Group I of ages under 14 (Supplementary Fig. S3D, *P* = 0.097), and Group II ages ≥ 20 (Supplementary Fig. S3I, *P* = 0.058). Adjusted hazard ratio (HR) of recurrence according to treatment group and gender were described in Table 4.

**Table 4:** Adjusted Hazard ratio after 1st episode of pneumothorax according to treatment groups and gender. Gray’s test was used for calculating subdistribution hazards.

Variables	Treatment				Gender				
	Group I (conservative)		Group II (surgery)		Male		Female		
	HR (95% CI)	p-value	HR (95% CI)	p-value	HR (95% CI)	p-value	HR (95% CI)		p-value
Age									
< 14	1.07 (0.99–1.16)	0.073	5.74 (5.12–6.42)	< 0.001	1.63 (1.52–1.74)	< 0.001	0.97 (0.81–1.15)		< 0.001
≥ 14, < 20	2.53 (2.46–2.61)	< 0.001	2.85 (2.72–2.99)	< 0.001	2.68 (2.62–2.75)	< 0.001	2.02 (1.87–2.18)		< 0.001
≥ 20	Ref		Ref		Ref		Ref		
Gender									
Male	Ref		Ref						
Female	0.59 (0.56–0.62)	< 0.001	0.74 (0.69–0.80)	< 0.001					
Treatment									
Group I (conservative)					Ref		Ref		
Group II (surgery)					1.16 (1.13–1.18)	< 0.001	1.57	(1.44–1.71)	< 0.001

*HR* Hazard ratio, *CI* confidential interval.

## Discussion

The incidence trends of spontaneous pneumothorax showed bimodal age-distribution, first peak at age between 15 to 34, and the other age beyond 60 s. Primary spontaneous pneumothorax usually involves young healthy population without pre-existing pulmonary diseases^1–3^. Spontaneous pneumothorax in elderly population is frequently associated with underlying chronic obstructive pulmonary disease (COPD), interstitial lung diseases or other pulmonary abnormalities therefore treatment process including prognosis could be differed; Preference of conservative treatment rather than surgery, and longer hospital stay as well as chest tube indwelling periods^27^. The goal of treatment in young spontaneous pneumothorax is to evacuate of air and if necessary, to prevent recurrence^8^. Surgery could be considered as first line treatment^12,28^.

A substantial proportion of PSP patients suffer from recurrence. In the relevant literature, there are reports of up to 50% recurrence after first events, mainly appearing within two to 24 months^4,20^. Surgical interventions are often recommended for prevention^12^, especially in cases in which bullae or blebs have been demonstrated on chest CT^29^. As a potential prevention, surgery may differ from the other therapeutic modalities that include observation, oxygen therapy, air aspiration, and catheter or chest tube insertion. These other modalities we describe as conservative management.

However, the protective effect of surgery has remained a controversial issue^8,20,30^. Previous studies have reported that surgery could be a risk factor for recurrence, not improving prognosis^14,30,31^. The recurrence rates in the young population who underwent surgery tended to increase^14^. In our study, with a few exceptions, PSP patients with surgical intervention experienced increased risk over those who had not been treated surgically. Only male patients aged ≥ 20 years showed no statistical significance between conservative and surgical management.

Notably, patients under 14 had a large discrepancy in recurrence rates between conventional and surgical treatment groups, regardless of gender. The overall 5-year recurrence rates were significantly higher in the ≥ 14 years, and < 20 years categories, and those of Group I showed similar trends. In Group II patients, those under 14 showed up to a 50% recurrence rate, nearly double that of other age categories.

A recent Korean study showed that the plateau of the pubertal growth spurt was at 14 years in girls and at 16 in boys^25,32^. The maximal growth velocity has tended to be faster^25^; therefore, 14 would be a better cutoff for Korean children to distinguish ages in volumetric growth from maturation periods. Age under 20 could suggest late physical maturation and end of adolescence. We assumed that patients under the growth spurt age were likely to be more susceptible than in maturation periods.

Several investigations have suggested that younger age, lower BMI, and delayed puberty could be risk factors for recurrence, and these findings are consistent with the results of this study^4,8,20^. Lower BMI was often observed in children prior to the pubertal growth spurt; children experienced increases in height earlier than weight gain. Delayed puberty was related to a postponed growth spurt because the acceleration of skeletal growth velocity was observed in the early stage of puberty^22^. In male patients aged ≤ 20, the recurrence rate was not significantly different. This finding was consistent with our hypothesis.

Our study revealed that the recurrence rates of female patients were significantly lower than those of males in all age categories (Supplementary Fig. S3A–C). These findings were quite different from previous studies that showed that the recurrence rates of females were significantly higher or had no association with gender. Female pneumothorax is related to gender-specific pulmonary abnormalities such as lymphangioleiomyomatosis or catamenial syndrome^4,30,33^. The exclusion of combined lung disease patients could have influenced our results.

The recurrence rate of a patient under the age of 14 who underwent conservative management was not statistically different between genders, but male patients who underwent surgical management showed higher recurrence rates (Supplementary Fig. S3D,G). This could suggest that developing lung parenchyma of a male experiencing a pubertal growth spurt tends to be more susceptible to mechanical damage. There are reported inconsistencies between growth in chest wall dimension and height increase in males, not in females^34^. Male growth is significantly greater, and male growth velocity is significantly faster^22^. This seemingly should be related to delayed maturation of the lung surface, resulting in fragility; however, no direct evidence of this has been reported^34^. Surgical intervention showed no statistically significant influence on recurrences between gender after adolescent periods in our study, and this could be indirect evidence for an inverse relationship between rapid growth and pleural strength.

Our study had several limitations. First, the reason for surgical intervention for enrolled patients was not clear. Usually, surgery could be recommended when the lung has not been expanded over 7 days, large bullae are noted, or there is a recurrence^5,8,20^. In our study, the surgery seemed to have occurred after the first PSP event. Although we tried to exclude secondary pneumothorax cases, other reasons needed for surgical intervention may remain. The results of our study could be significantly limited due to the potential selection bias stemmed from data extraction process. Health insurance claim data provide medical service use information under specific diagnostic codes and follow-up statistics, however, it does not secure the legitimacy of each treatment process. The lack of information relating with radiologic or pathologic findings essential for treatment strategies could cause severe skewedness in study materials, and therefore lead to inevitable restriction in data interpretation of our study.

Second, precocious characteristics such as height, weight, BMI, annual growth velocity, or family history can affect the recurrence rate^4,15,31,33^. We could not identify anthropometric variables, which can be related to recurrence. Finally, conventional treatment comprised a broad spectrum of therapeutic options, from simple observation to large bore chest tube insertion in this study. This lack of homogeneity among treatments could critically affect recurrence, but our results did not reflect these factors. Further evaluation for each treatment modality using a large patient population is necessary.

Limitations could be related to a lack of understanding of the air leak mechanism details. Although the presence of ELCs, bullae or blebs, could justify early surgical intervention for the prevention of recurrences^12^, the traditional concept as being main sources for occurrence makes this doubtful^8,30^. Notable pathologic lesions have not been reported in certain patients^18^, and the collateral ventilation system among pulmonary lobes has not been fully comprehended^30^.

Our study showed significant association between higher recurrence rates and surgical intervention in young PSP patients who did not complete their physical growth (children, preadolescence and adolescence). This does not guarantee that that surgery is a negative prognostic factor for recurrence because of significant possibility in data skewedness, however, increasing interest in less invasive treatment strategies for primary spontaneous pneumothorax^23,35,36^, serials of various conservative management could be considered before determination of surgery.

## Conclusion

Higher recurrence rates after 1st episodes of PSP were significantly associated with adolescence, male and surgery. Patients of children and preadolescence in surgery group showed over 50% of recurrence rates. Adolescent patient showed higher recurrence both in conservative and surgical groups, however, the latter was significantly higher than the former. Incompleteness of physical growth could be related with higher recurrence of pneumothorax. The differences in recurrence rates in surgical group seemed to be decrease when the patients are in adult periods. Enrolled data from NHIS did not contain precious individual differences such as BMI, the size of detected bullae or necessity for surgical intervention, results should be considered carefully.

### Supplementary Information

Supplementary Information 1.

Supplementary Information 2.

Supplementary Information 3.

Supplementary Information 4.

## Data Availability

The authors confirm that the data supporting the findings of this study are available within the article and its supplementary table and figures.

## Abbreviations

NHIS: National Health Insurance Service
PSP: Primary spontaneous pneumothorax
ELCs: Emphysema-like changes
IRB: Institutional review board
ICD: International Statistical Classification of Diseases and Related Health problems
BMI: Body mass index
HR: Hazard ratio
CI: Confidential interval
