# Supplementary figures and images for: Trends in recurrence of primary spontaneous pneumothorax in young population after treatment for first episode based on a nationwide population data
Source: Sci Rep. 2023 Aug 18;13:13478. doi: 10.1038/s41598-023-39717-y (PMC10439191; doi:10.1038/s41598-023-39717-y)

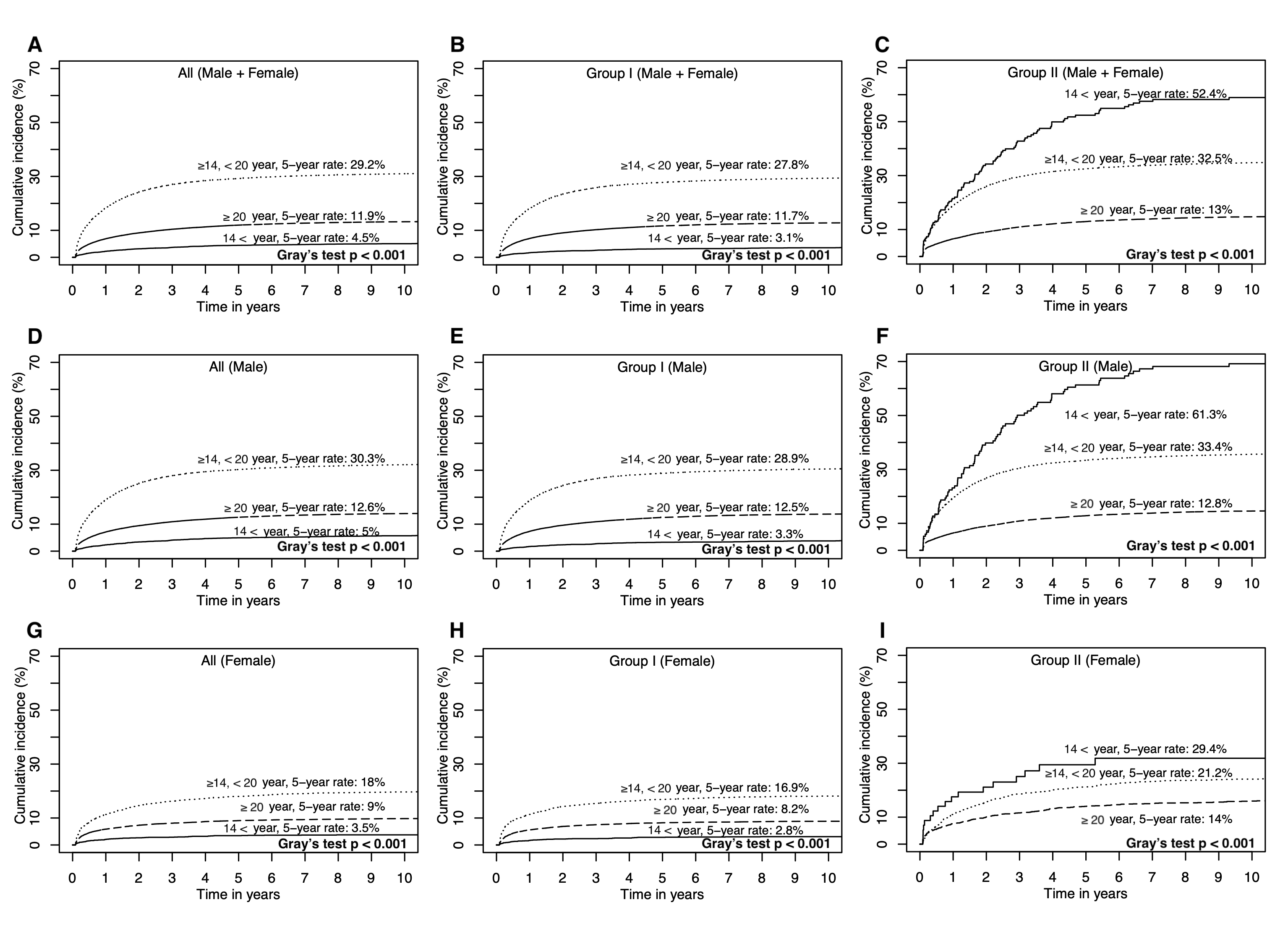

Supplement: Supplementary file 1 — Supplementary Information 1. [file 41598_2023_39717_MOESM1_ESM.tiff]

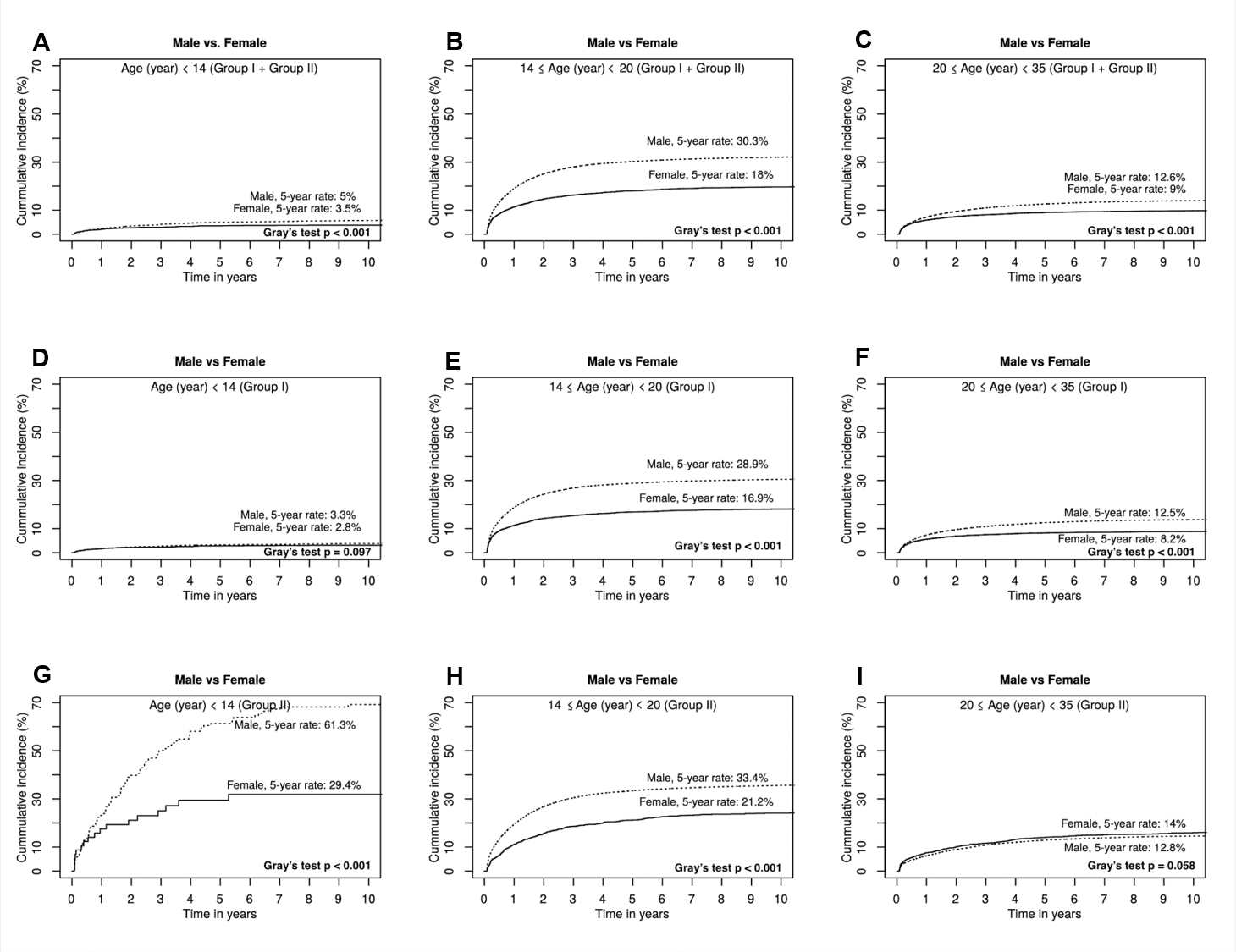

Supplement: Supplementary file 2 — Supplementary Information 2. [file 41598_2023_39717_MOESM2_ESM.tif]
