# Supplementary material for: Trends in recurrence of primary spontaneous pneumothorax in young population after treatment for first episode based on a nationwide population data
Source: Sci Rep. 2023 Aug 18;13:13478. doi: 10.1038/s41598-023-39717-y (PMC10439191; doi:10.1038/s41598-023-39717-y)
Supplement: Supplementary file 3 — Supplementary Information 3. [file 41598_2023_39717_MOESM3_ESM.docx]

**Supplementary Table 1**. Excluded ICD codes

| [A15.0] | Tuberculosis of lung, confirmed by sputum microscopy with or without culture |
| --- | --- |
| [A15.00] | Tuberculous of lung with cavitation, confirmed by sputum microscopy with or without culture |
| [A15.01] | Tuberculous of lung without cavitation or unspecified, confirmed by sputum microscopy with or without culture |
| [A15.1] | Tuberculosis of lung, confirmed by culture only |
| [A15.10] | Tuberculosis of lung with cavitation, confirmed by culture only |
| [A15.11] | Tuberculosis of lung without cavitation or unspecified, confirmed by culture only |
| [A15.2] | Tuberculosis of lung, confirmed histologically |
| [A15.20] | Tuberculosis of lung with cavitation, confirmed histologically without culture |
| [A15.21] | Tuberculosis of lung without cavitation or unspecified, confirmed histologically |
| [A15.3] | Tuberculosis of lung, confirmed by unspecified means |
| [A15.30] | Tuberculosis of lung with cavitation, confirmed by unspecified means |
| [A15.31] | Tuberculosis of lung without cavitation or unspecified, confirmed by unspecified means |
| [A16.0] | Tuberculosis of lung, bacteriologically and histologically negative |
| [A16.00] | Tuberculosis of lung with cavitation, bacteriologically and histologically negative |
| [A16.01] | Tuberculosis of lung without cavitation or unspecified, bacteriologically and histologically negative |
| [A16.1] | Tuberculosis of lung, bacteriological and histological examination not done |
| [A16.10] | Tuberculosis of lung with cavitation, bacteriological and histological examination not done |
| [A16.11] | Tuberculosis of lung without cavitation or unspecified, bacteriological and histological examination not done |
| [A16.2] | Tuberculosis of lung, without mention of bacteriological or histological confirmation |
| [A16.20] | Tuberculosis of lung with cavitation, without mention of bacteriological or histological confirmation |
| [A16.21] | Tuberculosis of lung without cavitation or unspecified, without mention of bacteriological or histological confirmation |
| [A20.2] | Pneumonic plague |
| [A24.1] | cute and fulminating melioidosis |
| [A40.3] | Sepsis due to Streptococcus pneumoniae |
| [A48.2] | Nonpneumonic Legionnaires’ disease [Pontiac fever] |
| [A50.0] | Early congenital syphilis, symptomatic |
| [A54.8] | Other gonococcal infections |
| [A56] | Other sexually transmitted chlamydial diseases |
| [A74] | Other diseases caused by chlamydiae |
| [B01.2] | Varicella pneumonia |
| [B05.2] | Measles complicated by pneumonia |
| [B06.8] | Rubella with other complications |
| [B20.6] | HIV disease resulting in Pneumocystis jirovecii pneumonia |
| [B22.1] | HIV disease resulting in lymphoid interstitial pneumonitis |
| [B25.0] | Cytomegaloviral pneumonitis |
| [B35-B49] | Mycoses |
| [B59] | Pneumonia due to Pneumocystis carinii(J17.3*) |
| [B95.3] | Streptococcus pneumoniae as the cause of diseases classified to other chapters |
| [B96.0] | Mycoplasma pneumoniae |
| [B96.1] | Klebsiella pneumoniae |
| [G00.1] | Pneumococcal meningitis |
| [G00.8] | Other bacterial meningitis |
| [I30.1] | Infective pericarditis |
| [J09] | Influenza due to identified zoonotic or pandemic influenza virus |
| [J09-J18] | Influenza and pneumonia |
| [J10] | Influenza due to identified seasonal influenza virus |
| [J10.0] | Influenza with pneumonia, seasonal influenza virus identified |
| [J11] | Influenza, virus not identified |
| [J11.0] | Influenza with pneumonia, virus not identified |
| [J12] | Viral pneumonia, NEC |
| [J12.0] | Adenoviral pneumonia |
| [J12.1] | Respiratory syncytial virus pneumonia |
| [J12.2] | Parainfluenza virus pneumonia |
| [J12.3] | Human metapneumovirus pneumonia |
| [J12.8] | Other viral pneumonia |
| [J12.80] | Human bocavirus pneumonia |
| [J12.88] | Other viral pneumonia |
| [J12.9] | Viral pneumonia, unspecified |
| [J13] | Pneumonia due to Streptococcus pneumoniae |
| [J14] | Pneumonia due to Haemophilus influenzae |
| [J15] | Bacterial pneumonia, NEC |
| [J15.0] | Pneumonia due to Klebsiella pneumoniae |
| [J15.1] | Pneumonia due to Pseudomonas |
| [J15.2] | Pneumonia due to staphylococcus |
| [J15.3] | Pneumonia due to streptococcus, group B |
| [J15.4] | Pneumonia due to other streptococci |
| [J15.5] | Pneumonia due to Escherichia coli |
| [J15.6] | Pneumonia due to other Gram-negative bacteria |
| [J15.7] | Pneumonia due to Mycoplasma pneumoniae |
| [J15.8] | Other bacterial pneumonia |
| [J15.9] | Bacterial pneumonia, unspecified |
| [J16] | Pneumonia due to other infectious organisms, NEC |
| [J16.0] | Chlamydial pneumonia |
| [J16.8] | Pneumonia due to other specified infectious organisms |
| [J17] | Pneumonia in diseases classified elsewhere |
| [J17.0] | Pneumonia in bacterial diseases classified elsewhere |
| [J17.1] | Pneumonia in viral diseases classified elsewhere |
| [J17.2] | Pneumonia in mycoses |
| [J17.3] | Pneumonia in parasitic diseases |
| [J17.8] | Pneumonia in other diseases classified elsewhere |
| [J18] | Pneumonia, organism unspecified |
| [J18.1] | Lobar pneumonia, unspecified |
| [J18.2] | Hypostatic pneumonia, unspecified |
| [J18.8] | Other pneumonia, organism unspecified |
| [J18.9] | Pneumonia, unspecified |
| [J20.0] | Acute bronchitis due to Mycoplasma pneumoniae |
| [J66] | Airway disease due to specific organic dust |
| [J67] | Hypersensitivity pneumonitis due to organic dust |
| [J67.1] | Bagassosis |
| [J67.8] | Hypersensitivity pneumonitis due to other organic dusts |
| [J67.9] | Hypersensitivity pneumonitis due to unspecified organic dust |
| [J68.0] | Bronchitis and pneumonitis due to chemicals, gases, fumes and vapours |
| [J69] | Pneumonitis due to solids and liquids |
| [J69.0] | Pneumonitis due to food and vomit |
| [J69.1] | Pneumonitis due to oils and essences |
| [J69.8] | Pneumonitis due to other solids and liquids |
| [J70.0] | Acute pulmonary manifestations due to radiation |
| [J81] | Pulmonary oedema |
| [J82.8] | Other pulmonary eosinophilia |
| [J84] | Other interstitial pulmonary diseases |
| [J84.10] | Lymphoid interstitial pneumonia |
| [J84.18] | Other interstitial pulmonary diseases with fibrosis |
| [J84.9] | Interstitial pulmonary disease, unspecified |
| [J85.1] | Abscess of lung with pneumonia |
| [J85.2] | Abscess of lung without pneumonia |
| [J95.4] | Mendelson’s syndrome |
| [M00.1] | Pneumococcal arthritis and polyarthritis |
| [M00.10] | Pneumococcal arthritis and polyarthritis, multiple sites |
| [M00.11] | Pneumococcal arthritis and polyarthritis, shoulder region |
| [M00.12] | Pneumococcal arthritis and polyarthritis, upper arm |
| [M00.13] | Pneumococcal arthritis and polyarthritis, forearm |
| [M00.14] | Pneumococcal arthritis and polyarthritis, hand |
| [M00.15] | Pneumococcal arthritis and polyarthritis, pelvic region and thigh |
| [M00.16] | Pneumococcal arthritis and polyarthritis, lower leg |
| [M00.17] | Pneumococcal arthritis and polyarthritis, ankle and foot |
| [M00.18] | Pneumococcal arthritis and polyarthritis, other |
| [M00.19] | Pneumococcal arthritis and polyarthritis, site unspecified |
| [O29.0] | Pulmonary complications of anaesthesia during pregnancy |
| [O74.0] | Aspiration pneumonitis due to anaesthesia during labour and delivery |
| [O89.0] | Pulmonary complications of anaesthesia during the puerperium |
| [P23] | Congenital pneumonia |
| [P23.0] | Congenital pneumonia due to viral agent |
| [P23.1] | Congenital pneumonia due to chlamydia |
| [P23.2] | Congenital pneumonia due to staphylococcus |
| [P23.3] | Congenital pneumonia due to streptococcus, group B |
| [P23.4] | Congenital pneumonia due to Escherichia coli |
| [P23.5] | Congenital pneumonia due to [P23.5]seudomonas |
| [P23.6] | Congenital pneumonia due to other bacterial agents |
| [P23.8] | Congenital pneumonia due to other organisms |
| [P23.9] | Congenital pneumonia, unspecified |
| [P24] | Neonatal aspiration syndromes |
| [P24.9] | Neonatal aspiration syndrome, unspecified |
| [P35-P39] | Infections specific to the perinatal period |
| [P35.0] | Congenital rubella syndrome |
| [T66] | Unspecified effects of radiation |
| [Q00-Q07] | Congenital malformations of the nervous system |
| [Q10-Q18] | Congenital malformations of eye, ear, face and neck |
| [Q20-Q28] | Congenital malformations of the circulatory system |
| [Q30-Q34] | Congenital malformations of the respiratory system |
| [Q35-Q37] | Cleft lip and cleft palate |
| [Q38-Q45] | Other congenital malformations of the digestive system |
| [Q50-Q56] | Congenital malformations of genital organs |
| [Q60-Q64] | Congenital malformations of the urinary system |
| [Q65-Q79] | Congenital malformations and deformations of the musculoskeletal system |
| [Q80-Q89] | Other congenital malformations |
| [Q90-Q99] | Chromosomal abnormalities, NEC |
