# Supplementary material for: Trends in recurrence of primary spontaneous pneumothorax in young population after treatment for first episode based on a nationwide population data
Source: Sci Rep. 2023 Aug 18;13:13478. doi: 10.1038/s41598-023-39717-y (PMC10439191; doi:10.1038/s41598-023-39717-y)
Supplement: Supplementary file 4 — Supplementary Information 4. [file 41598_2023_39717_MOESM4_ESM.docx]

**Supplementary Figure S2.** **Recurrence curves according to the age categories in all patient and each gender groups. the 5-year recurrence rates of age category ≤14 and <20 were significantly higher than other age categories when compared in all patients (A), in Group I (B) and Group II (C). When analyzing male and female patients separately (D to I), age category ≤14 and <20 showed significant higher recurrence rates than other age groups except Group II of male. Age category under 14 showed higher recurrence rates (61.3%), which showed statistical significance among other age categories (F). A. Recurrence curves according to the age categories All patients (same curve of Figure3D). B. Recurrence curves of Group I, and C. Group II. D. Recurrence curves in male patients according to the age categories. E. Those of Group I and, F. Group II in male patients. G. Recurrence curves in Female patients according to the age categories. H. Those of Group I and, I. Group II in female patients.**

**Supplementary Figure S3**. Comparison of 5-year recurrence rates between male and female according to age categories and treatment groups. Male patients showed significantly higher recurrence rates in all age and treatment groups except age categories <14 of Group I and ≥20 of Group II. A. The 5-year recurrence curves of male and female in patients under aged 14. B. Those in aged ≤ and >20. C. Those in aged ≥20. D. Comparison of 5-year recurrence rates between male and female in patients under aged 14 of Group I. E. Those in aged ≤ and >20. F. Those in aged ≥20. G. Comparison of 5-year recurrence rates between male and female in patients under aged 14 of Group II. H. Those in aged ≤ and >20. I. Those in aged ≥20.
